# Evaluation of P-glycoprotein-targeting circulating microRNAs as peripheral biomarkers for medically intractable epilepsy

**DOI:** 10.1186/s42494-022-00116-x

**Published:** 2023-01-23

**Authors:** Yangmei Xie, Yiye Shao, Xue Gong, Ming Wang, Yinghui Chen

**Affiliations:** 1 Department of Neurology, Huashan Hospital, Fudan University, National Center for Neurological Disorders, 12 Wulumuqi Road, Shanghai, 200040 China; 2 Department of Neurology, Shanghai General Hospital, Shanghai Jiao Tong University School of Medicine, Shanghai, 200940 China; 3 Community Health Service Center of Xinqiao Town, Songjiang District, Shanghai, 201612 China

**Keywords:** Intractable epilepsy, Drug resistance, P-glycoprotein, Circulating miRNAs, Biomarker

## Abstract

**Background:**

Early diagnosis of medically intractable epilepsy is challenging in clinical work. P-glycoprotein (P-gp) is one of the most important multidrug efflux transporters, which has been demonstrated to contribute to the drug resistance of intractable epilepsy. The present study was aimed to explore the diagnostic value of microRNAs (miRNAs) targeting P-gp for medically intractable epilepsy.

**Methods:**

Thirty-six patients with intractable epilepsy and 36 epilepsy patients responsive to anti-epilepsy drugs, who visited Jinshan Hospital of Fudan University from September 2014 to September 2016, were enrolled in this study. Clinical information of the patients was obtained by retrospectively reviewing medical records. MiRNAs with differential serum expression between the two groups of patients were detected by microarray assay. Meanwhile, miRNAs that were confirmed to regulate P-gp in vitro by western blot were selected for further validation. In the validation phase, reverse transcription quantitative PCR (RT-qPCR) was conducted to confirm the differential expression of the candidate miRNAs in the epilepsy cohorts. Receiver operating characteristic (ROC) curve analysis was carried out to evaluate the diagnostic value of the miRNAs for intractable epilepsy.

**Results:**

Three miRNAs including miR-6514-3p, miR-6076-5p, and miR-6855-3p were identified to be candidate miRNAs by microarray assay. The results of western blotting validated that miR-146a-5p and miR-138-5p could regulate P-gp expression in vitro, so they were included in the candidate miRNAs for further validation. In the validation phase, the results of RT-qPCR indicated that compared with drug-responsive patients, the patients with intractable epilepsy showed decreased level of miR-138-5p and increased level of miR-146a-5p. The results of ROC curve analysis indicated that miR-138-5p (AUC = 0.877) and miR-146a-5p (AUC = 0.866) had high diagnostic value for intractable epilepsy. In addition, the miR-panel composed of miR-138-5p and miR-146a-5p showed higher diagnostic value (AUC = 0.926) than the miRNAs selected by microarray assay.

**Conclusions:**

Our results indicated that the dysregulated miR-138-5p and miR-146a-5p which target P-gp expression have high potential as peripheral biomarkers for medically intractable epilepsy.

**Supplementary Information:**

The online version contains supplementary material available at 10.1186/s42494-022-00116-x.

## Background

Epilepsy is one of the most common neurological disorders. Currently, there are about 60 million people with epilepsy worldwide, with 5 million new cases every year [1]. Approximately 30% of patients exhibit poor efficacy of antiseizure medications (ASMs) and eventually evolve into medically intractable or drug-resistant epilepsy. However, current diagnosis of medically intractable epilepsy is mainly based on long-term clinical observation of curative effects of ASMs, which may cause poor prognosis and bring a huge economic burden to the family and the whole society [2]. In recent decades, drug resistance has become a great challenge for epilepsy treatment. Therefore, early diagnosis of medically intractable epilepsy is critically important for application of appropriate treatments while avoiding serious complications. However, there is a lack of reliable markers for early diagnosis at present, especially markers in peripheral blood.

MicroRNAs (miRNAs) are a class of endogenous short noncoding RNAs of approximately 22–25 nt in length, which can post-transcriptionally modulate gene expression [3]. Emerging studies have implied that miRNAs are involved in the occurrence and development of epilepsy [4, 5]. In the human body, miRNAs are secreted from specific tissues and circulate to the peripheral blood, functioning in information transmission. Previous studies have demonstrated that miRNAs are abundant and stable in the peripheral blood, and can tolerate different temperatures, pH conditions, repeated freezing and thawing [6]. In addition, detection of miRNAs in the peripheral blood is rapid, noninvasive and economical [7]. Thus, the circulating miRNAs are emerging as promising biomarkers for various diseases, especially cancer. Recently, several studies have explored the potential value of the circulating miRNA as a diagnostic marker for medically intractable epilepsy. One study found that the level of miR-633b in the serum is increased gradually from 30 min to 2 days after epilepsy onset, while decreased to the baseline 2 days later, which indicated that the miRNA abundance in serum is closely associated with the state of epileptic seizures [8]. Another study implied that the level of miR-145-5p in the serum of patients with medically intractable epilepsy is closely associated with the age of epilepsy onset, the epilepsy history and the seizure frequency [9]. Additionally, the level of miR-301a-3p is negatively correlated with seizure severity in medically intractable epilepsy, which suggests a high diagnostic value of miR-301a-3p for drug-resistant epilepsy [10]. However, these investigations focused on the dysregulation of miRNAs, but rarely correlated miRNA dysregulation with the pathogenesis of medically intractable epilepsy. In fact, the expression profiles of miRNAs in the serum can be influenced by multiple factors, as miRNAs participate in many biological processes such as cell proliferation, apoptosis, differentiation, and metabolism [11]. Therefore, the method of screening miRNA biomarkers for medically intractable epilepsy merely based on serum microarray may lack specificity.

In fact, the pathogenesis of medically intractable epilepsy is complicated, involving both genetic and environmental factors [12]. P-glycoprotein (P-gp) is one of the most important multi-drug efflux transporters in the brain, which can transport conventional ASMs such as phenytoin (PHT) and phenobarbital [13]. Many studies have demonstrated that the expression of P-gp is significantly upregulated in the brain of patients and animal models of intractable epilepsy, which may limit the delivery of ASMs to the epileptic brain tissue [14], while downregulation of P-gp could increase the cerebral concentration of ASMs and improve the therapeutic effect for epilepsy [15]. Therefore, overexpression of P-gp has become a potential biomarker to discriminate between drug-responsive epilepsy and drug-resistant epilepsy. However, P-gp is mainly expressed at the microvascular endothelium in the central nervous system and rarely released into the peripheral blood, making it hard to detect P-gp directly in vivo. Identifying endogenous molecules that modulate P-gp in the peripheral blood may provide an alternative strategy. Our previous study has demonstrated that miRNAs could increase ASM accumulation in the brain of rats with intractable epilepsy by regulating P-gp expression [16]. In this study, we compared the diagnostic value of candidate miRNAs identified by microarray assay and candidate miRNAs targeting P-gp, in order to explore potential circulating biomarkers for medically intractable epilepsy.

## Materials and methods

### Study design and patients

According to the criteria proposed by the International League Against Epilepsy in 2010 [17], 36 patients diagnosed with intractable epilepsy and 36 patients diagnosed with with drug-resistent epilepsy, who visited department of Neurology at Jinshan Hospital of Fudan University from September 2014 to September 2016 were enrolled in this study. All the patients received comprehensive clinical examinations, including the a review of medical history, physical and psychiatric examination, laboratory examination, and electroencephalogram (EEG) recording. All patients were evaluated by two trained neurologists using standardized questionnaires for general information including seizure history, seizure frequency, and ASM treatment. This study was approved by the local medical ethics committee and carried out in accordance with the approved guidelines issued by ethics committee of Jinshan Hospital of Fudan University. Written informed consent was obtained from all patients or their close relatives. Drug-responsive epilepsy was defined as epilepsy patients responsive to ASM treatment and remaining seizure-free for more than 1 year before blood collection. Medically intractable epilepsy or drug-resistant epilepsy was defined as patients with epilepsy suffering from seizures more than 4 times per month despite treatment with appropriate ASMs for 2 years. Patients with a history of hematological system disorder, autoimmune disease, immune deficiency disease, stroke, severe cognitive impairment, tumor, or central infection 2 weeks before sample collection were excluded from this study.

### Blood sample collection

A total of 5 ml blood was collected from each patient, and then centrifuged at 4000 g for 5 min at room temperature to obtain the serum, within 1 h after blood collection [7]. The serum samples were stored in RNase-free tubes at − 80 °C until use.

### RNA extraction and reverse transcription quantitative PCR (RT-qPCR)

Total RNA was extracted from serum using the TRIzol Reagent (Invitrogen Life Technologies, Carlsbad, CA, USA) according to the manufacturer’s instructions. The purity and the concentration of RNA were determined using NanoDrop Lite Spectrophotometer (Thermo, Germany). Then the total RNA was reversely transcribed into cDNA and amplified using the miRCURY LNA™ Universal RT microRNA PCR system (Exiqon, Denmark) in Gene Amp PCR System 9700 (Applied Biosystems, Foster city, CA, USA). The expression of miRNAs in the serum was normalized to miR-93-5p and calculated using the 2^ − ΔΔCt^ method.

### Microarray analysis of serum miRNA

The differentially expressed miRNAs in the serum were screened by Agilent microarray analysis at the Beijing Bioassay Laboratory of CapitalBio Corporation (Beijing, China). RNA was labeled with the Agilent miRNA labeling reagent and then hybridized to gene chips. The gene chips were scanned with the Agilent microarray scanner (Agilent) and data were processed using the Agilent feature extraction software version 10.10. The criteria for significant differentially expressed miRNAs were *P* < 0.05 and fold change > 2.0.

### Cell culture and transient transfection

Human brain microvascular endothelial cells (HBMECs) were purchased from ScienCell Research Laboratories (Carlsbad, CA). Drug-resistant HBMECs were induced by repetitive exposure to PHT continuously for 2 weeks as previously described [18]. The drug-resistant HBMECs were transfected with miRNA mimics or negative-control miRNA (miR-NC) using X-tremeGENE Transfection Reagent (Roche, Basel, Switzerland) according to the manufacturer’s instructions. The cells were collected 48 h after transfection for further use. The miRNA mimics and miR-NC were synthetized by RiboBio (Guangzhou, China).

### Western blot analysis

Total proteins were extracted from cells with lysis buffer (Beyotime, Shanghai, China).

The protein (20 μg) was separated by SDS-PAGE and transferred to a PVDF membrane. Then the membranes were incubated at 4 °C overnight with primary antibodies including anti-P-gp, anti-p-NF-κB p65, anti- NF-κB p65 and anti-GAPDH (Cell Signaling Technology, USA) in 5% non-fat milk. The membranes were washed with TBST and incubated with peroxidase-conjugated antibodies at 4 °C for 2 h. The protein bands were developed using the ECL-Plus kit (Merck Millipore, Germany), and the band intensity was quantified using ImageJ software.

### Statistical analysis

Statistical analyses were performed using SPSS 24.0 (SSPS, Inc., Chicago, IL). Values are expressed as the mean ± SD, and statistical significance was defined as *P* < 0.05. Clinical characteristics were compared using the chi-square test or the *t*-test. Expression of miRNAs was compared using the Kruskall-Wallis test or the Mann-Whitney U test. Receiver operator characteristic (ROC) curves were used to evaluate the diagnostic value of serum miRNAs for medically intractable epilepsy.

## Results

### Clinical characteristics of patients with epilepsy

A total of 72 participants (including 36 patients with drug-resistant epilepsy and 36 patients with drug-responsive epilepsy) were recruited in this study. There was no significant difference in age and gender between the two groups (*P* = 0.776 and 0.992). The duration of epilepsy in patients with drug-resistant epilepsy was significantly longer than that in patients with drug-responsive epilepsy (*P* < 0.05). The patients with drug-resistant epilepsy suffered more frequent seizures, and showed a higher proportion of polypharmacy at the last clinic visit, compared to the patients with drug-responsive epilepsy. The detailed clinical characteristics of patients are listed in Table 1.

**Table 1:** Clinical characteristics of patients with epilepsy

General information	Drug-responsive epilepsy	Drug-resistant epilepsy	*P* value
*n*	36	36	
Age, mean ± SD (years)	38.19 ± 10.93	38.64 ± 10.41	0.776
Gender (male: female)	18:18	19:17	0.992
Epilepsy duration, median (years)	3.33 (2-10)	9.67 (3-30)	*P* < 0.05
Seizure frequency, median (years)	2 (0-3)	84 (48-120)	*P* < 0.05
AED therapy at last visit			
Monopharmacy	26 (72.2%)	4 (11.1%)	*P* < 0.05
Polypharmacy	10 (27.8%)	32 (88.9%)	*P* < 0.05

### Differentially expressed miRNAs in the serum of patients with epilepsy

**Fig. 1:**
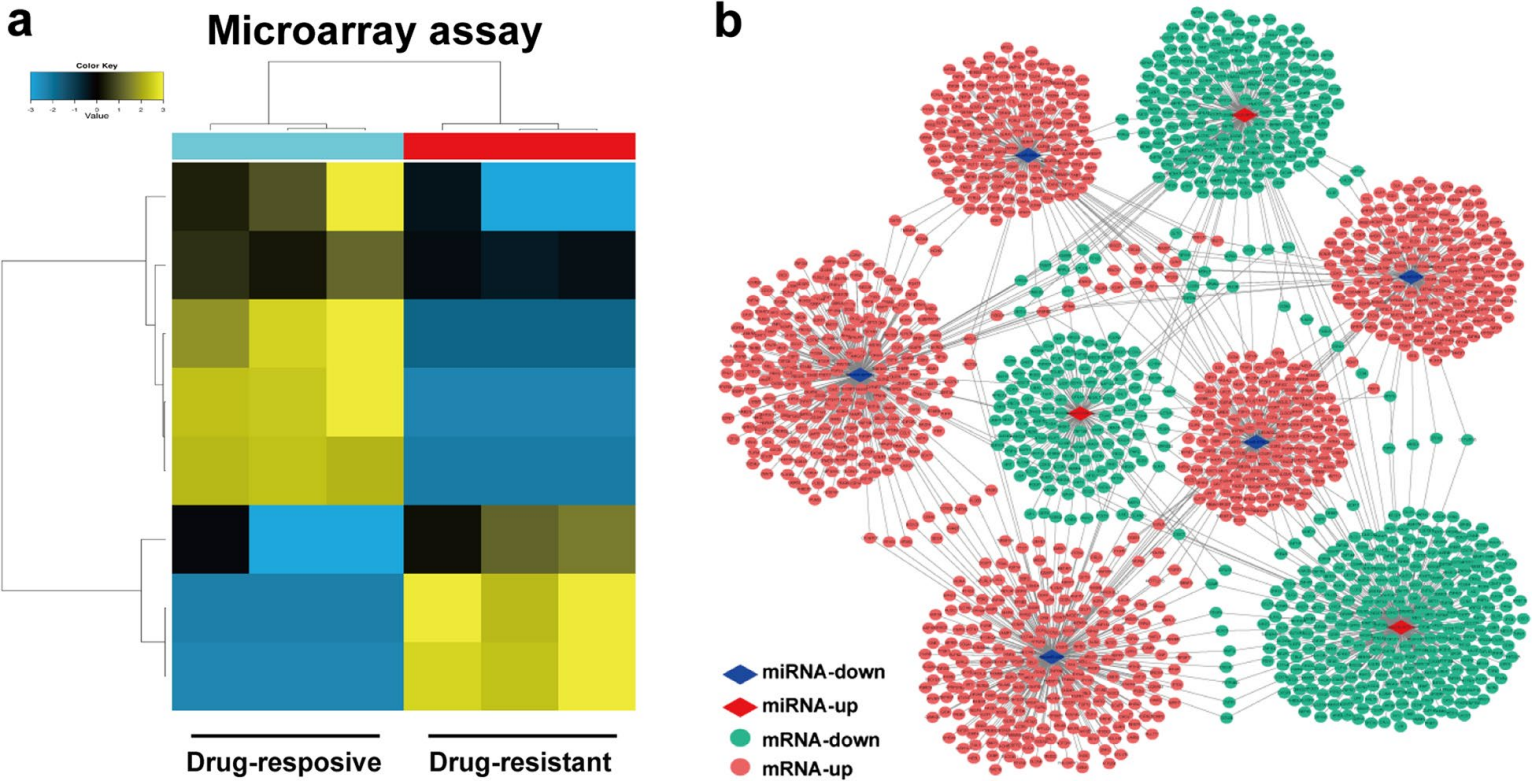
The miRNA profiling in the serum of patients with epilepsy by miRNA microarray. **a** A hierarchical cluster map of differentially expressed miRNAs in the serum of patients with drug-resistant epilepsy versus drug-responsive epilepsy. **b** Correlations between the miRNAs and their target genes as shown in the miRNA-mRNA network diagram

Microarray assay revealed a total of eight differentially expressed miRNAs between the two groups, as shown in the hierarchical cluster map (Fig. 1a). Compared with patients with drug-responsive epilepsy, three miRNAs were upregulated,including miR-6514-3p, miR-6873-3p and miR-3591-3p; while five miRNAs were downregulated, including miR-6076-5p, miR-6879-5p, miR-6855-3p, miR-6789-3p and miR-4476 in the serum of patients with drug-resistant epilepsy. The target genes of the differentially expressed miRNAs were predicted by three online software programs including miRanda, miRwalk and TargetScan. The target genes of the three prediction programs were integrated, and the intersection was selected. Finally, 1841 target genes of the eight dysregulated miRNAs were included to generate the network diagram of miRNA-mRNA correlations (Fig. 1b).

### Functional annotation of differentially expressed serum miRNAs

**Fig. 2:**
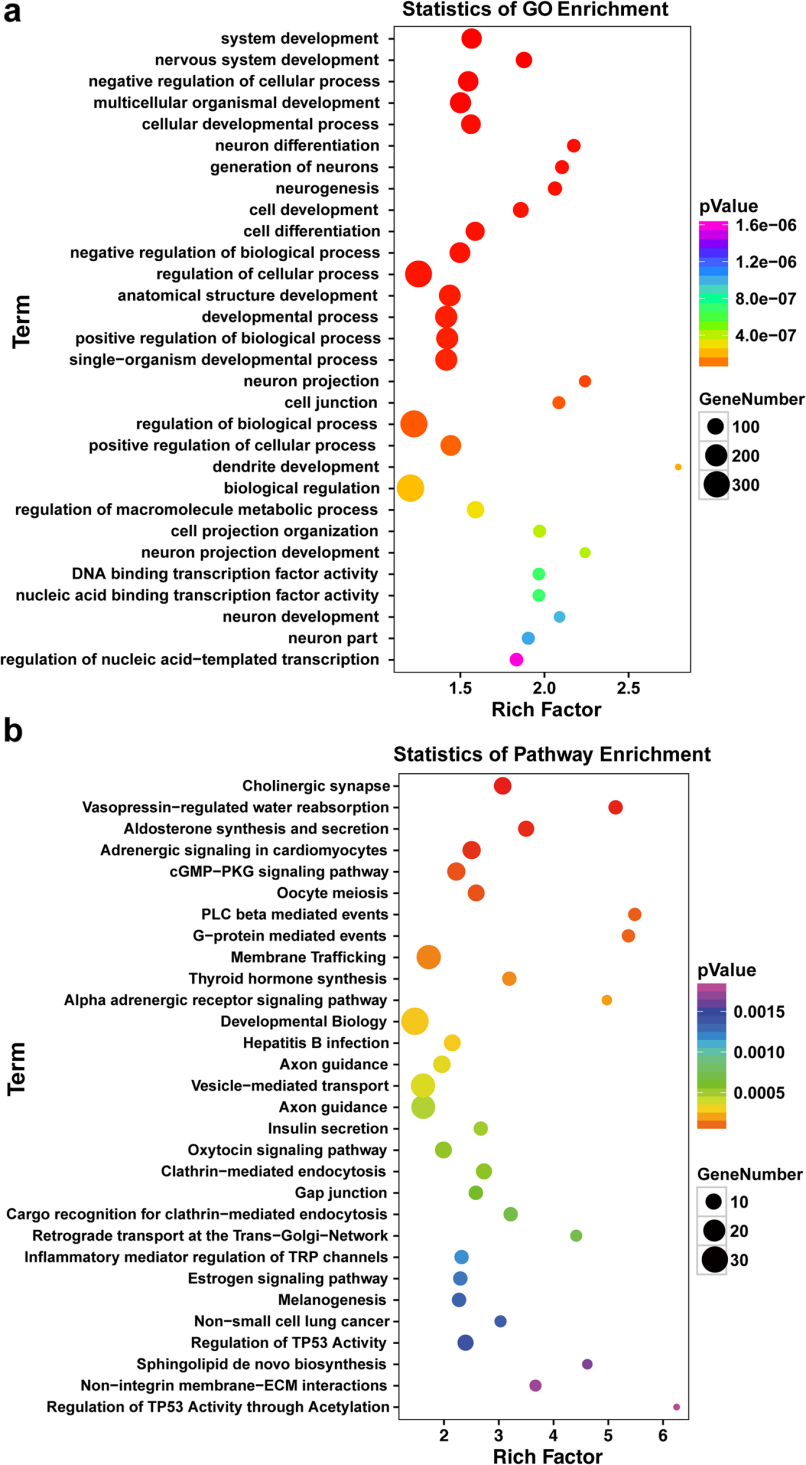
Functional annotation of target genes of the differentially expressed miRNAs in the serum of patients with epilepsy. **a** GO enrichment annotation of target genes of the differentially expressed serum miRNAs. **b** KEGG pathway analysis of target genes of differentially expressed serum miRNAs

The Gene Ontology (GO) and Kyoto Encyclopedia of Genes and Genomes (KEGG) analyses were performed for the target genes of the differentially expressed miRNAs using the online analysis tool (https://david.ncifcrf.gov/). The top 30 statistically significant GO terms are shown in Fig. 2a. The dysregulated miRNAs were involved in various biological processes, especially nervous system development, such as neuron differentiation, dendritic development, and neuron projection development. The top 30 statistically significant KEGG pathway terms are shown in Fig. 2b. The results indicated that the dysregulated miRNAs were associated with membrane trafficking, vesicle-mediated transport, and axon guidance.

### Identification of miRNAs regulating P-gp in vitro

**Fig. 3:**
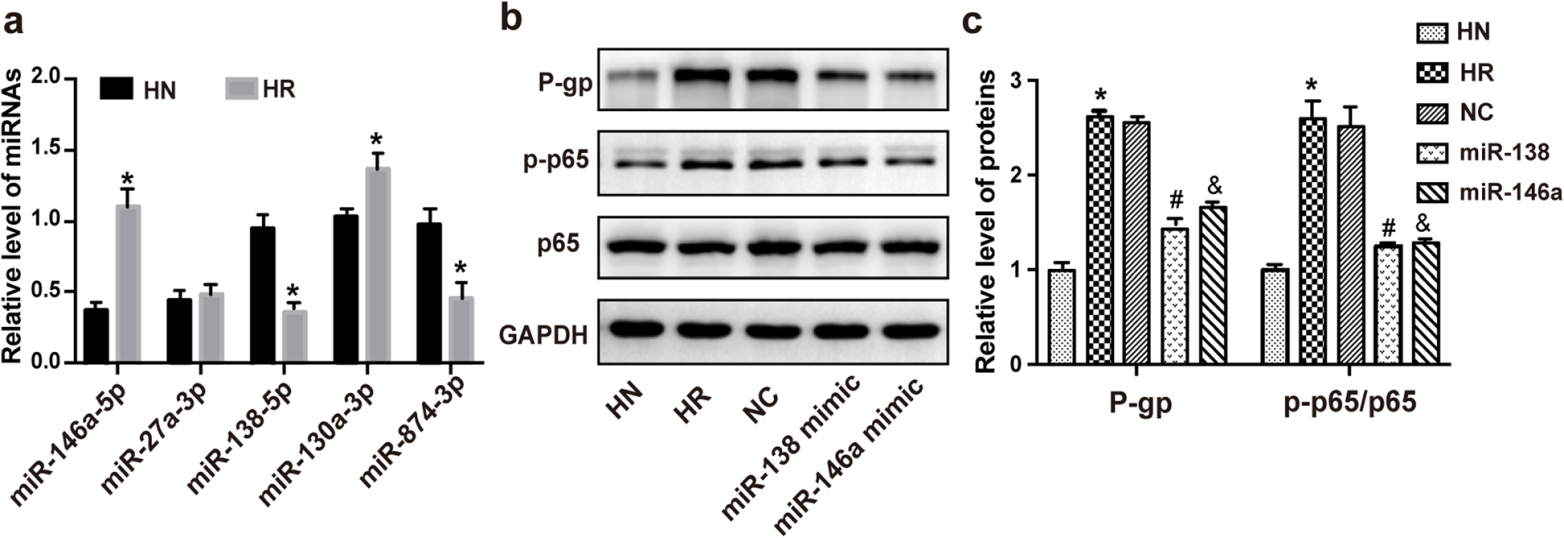
Identification of miRNAs targeting P-gp in vitro. **a** The expression of miRNAs in drug-resistant HBMECs and its parental cells. **b** Representative bands of P-gp, and key molecules of the NF-κB signaling pathway (p65/p-p65) in different groups. **c** Analysis of relative expression of P-gp and the activation of the NF-κB signaling pathway (p-p65/p65). HN, HBMEC; HR, HBMEC/PHT; NC, HBMEC/PHT transfected with negative control. *P < 0.05 vs HN, ^#^*P* < 0.05 vs HR, ^&^*P* < 0.05 vs HR

Overexpression of P-gp in drug-resistant HBMECs was induced by repetitive exposure to PHT. In our previous study, we detected a total of 92 miRNAs that were differentially expressed under antiepileptic drug resistance, using miRNA microarray [19]. In this study, we selected five miRNAs from them for further examination, including miR-146a-5p, miR-138-5p, miR-130a-3p, miR-27a-3p and miR-874-3p, which were probably associated with P-gp expression. The results of RT-qPCR indicated that the levels of miR-874-3p and miR-138-5p were downregulated while miR-146a-5p and miR-130a-3p were upregulated in drug-resistant HBMECs compared to the control HBMECs (Fig. 3a). The results of western blot analysis showed that overexpression of miR-138-5p and miR-146a-5p inhibited P-gp expression in the drug-resistant HBMECs (Fig. 3b). In addition, overexpression of miR-138-5p and miR-146a-5p suppressed activation of the classical regulator of P-gp, nuclear factor-kappaB (NF-κB) (p-p65/p65) (Fig. 3c).

### Validation of expression of candidate miRNAs in large-scale epilepsy patients

**Fig. 4:**
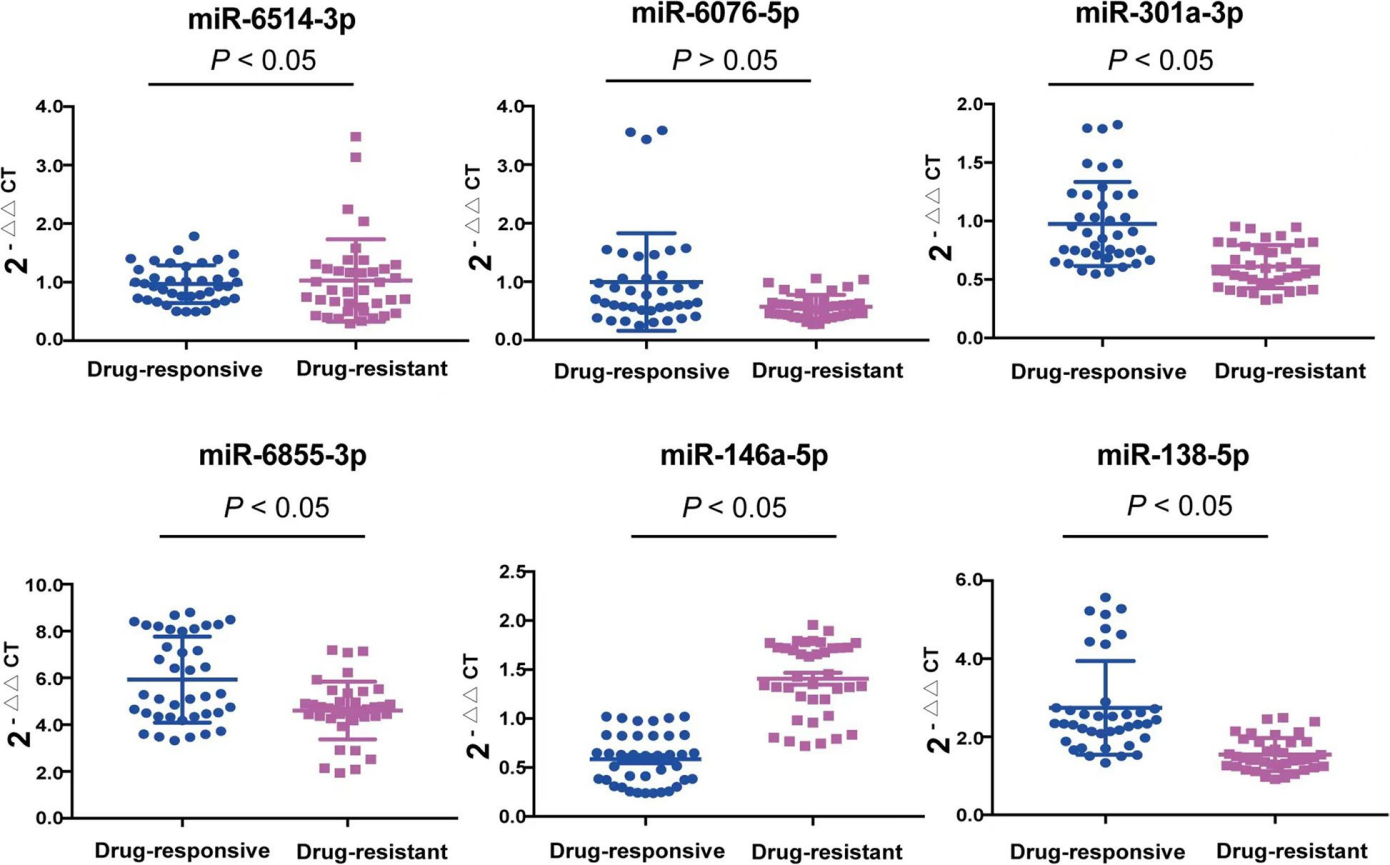
Validation of the six miRNAs selected in the discovery phase in drug-resistant epilepsy compared to drug-responsive epilepsy

The candidate miRNAs were selected based on the results of differentially expressed miRNAs detected by microarray assay and miRNAs associated with P-gp expression. Additionally, a previous study has reported that miR-301a-3p has a high diagnostic value for drug-resistant epilepsy [10], so we included it in the candidate miRNAs in this study. Finally, six miRNAs including miR-6514-3p, miR-6076-5p, miR-301a-3p, miR-6855-3p, miR-146a-5p and miR-138-5p were selected for further validation in the discovery phase. In the validation phase, the results of RT-qPCR indicated that compared with the drug-responsive epilepsy, the levels of miR-6855-3p, miR-138-5p and miR-301a-3p were decreased, while the levels of 6514-3p and miR-146a-5p were increased in patients with drug-resistant epilepsy. However, no significant difference was detected in miR-6076-5p expression level between the two groups of patients (Fig. 4).

### Evaluation of the diagnostic value of candidate miRNAs using the ROC model

**Fig. 5:**
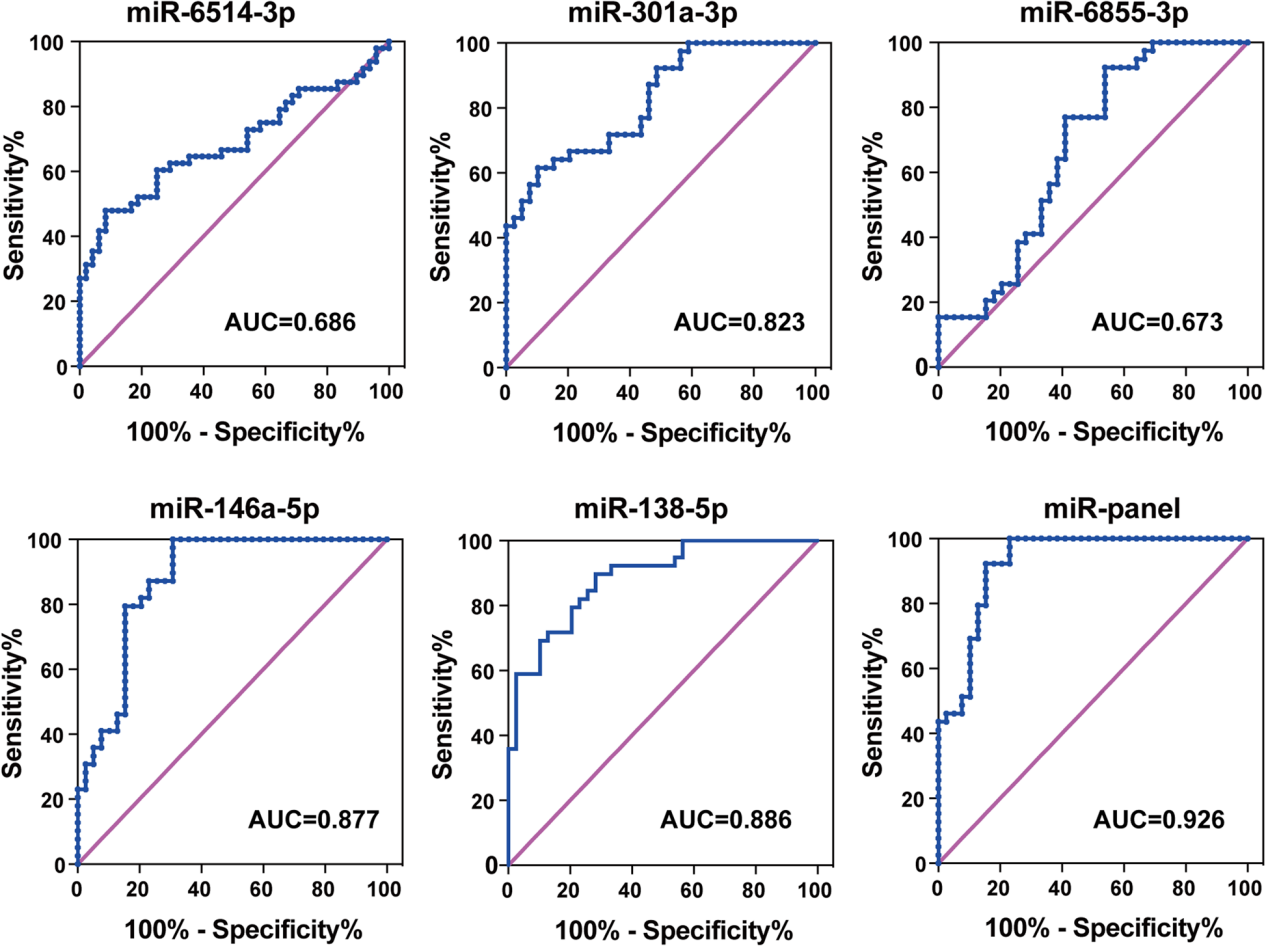
Evaluation of the diagnostic value for candidate miRNAs. Receiver operating characteristic (ROC) curve analysis of five candidate miRNAs selected in large-scale epilepsy patients and the miRNA panel for discriminating drug-resistant epilepsy from drug-responsive epilepsy. AUC, area under the ROC curve

ROC curve analysis indicated that among the candidate miRNAs, miR-138-5p and miR-146a-5p had AUC values higher than 0.85. Additionally, the AUC value of the miR-panel composed of miR-138-5p and miR-146a-5p was 0.926, showing a potential value as a peripheral biomarker of medically intractable epilepsy. However, the AUC values of miR-301a-3p, miR-6855-3p and miR-6514-3p were 0.826, 0.673 and 0.686, respectively (Fig. 5).

## Discussion

Currently, the diagnosis of medically intractable epilepsy mainly relies on clinical manifestations and medical history information, accompanied by auxiliary imaging examinations like MRI and PET [20]. However, most patients with epilepsy fail to accurately describe their symptoms and medical history, which makes the diagnosis quite difficult. Thus, reliable biomarkers are urgently needed for early diagnosis of medically intractable.

Present studies on biomarkers of medically intractable epilepsy mainly focused on diagnostic biomarkers in the cerebrospinal fluid and peripheral blood, brain imaging, and EEG recordings [21]. Accumulating evidence shows that the increased expression of P-gp in the brain is emerging as a potential molecular marker for medically intractable epilepsy [22]. A recent case cohort study using PET showed that the activity of P-gp in the brains of patients with medically intractable epilepsy is significantly higher than that in patients with drug-responsive epilepsy [14]. Compared with brain imaging, measurement of molecular markers in fluids are more objective, economic and practical. In recent years, multiple studies have found that P-gp expression is increased in the serum of patients with medically intractable epilepsy [23]. However, previous studies have demonstrated that P-gp is mainly expressed in the blood-brain barrier in patients with medically intractable epilepsy, rarely released into the blood. Additionally, the level of P-gp in peripheral blood may be influenced by other diseases like autoimmune diseases, hematological system disorders and tumors [24, 25]. Thus, the level of P-gp in the blood could hardly represent the level of P-gp in the brain.

MiRNAs are emerging promising diagnostic biomarkers of various diseases due to their tissue specificity, stability and high abundance in peripheral blood [26]. The circulating miRNAs in the serum are mainly from three sources: extracellular vesicle secretion packages, destructed tissues or lytic cells, and RNA-binding protein. MiRNAs can carry information associated with specific diseases and contribute to diagnosis of several diseases [27]. Previous studies have demonstrated that the circulating miRNAs have high clinical value as an auxiliary diagnosis index in cancer [28]. Recently, some studies have explored the potential of circulating miRNAs as diagnostic biomarkers for intractable epilepsy. For example, a previous study indicated that miR-4521 can be used as a peripheral marker for medically intractable epilepsy caused by cortical dysplasia [29]. In addition, several studies have shown that the sensitivity and specificity of miR-8071 reached 80% for diagnosis of temporal lobe epilepsy with hippocampal sclerosis [30]. These diagnostic biomarkers are only suitable for patients with medically intractable epilepsy induced by specific pathological lesions. However, in clinical practice, most intractable epilepsy patients have no clear lesions in pathological or MRI examinations. Thus, these approaches may have limited applicability. Accumulating evidence indicates that the overexpression of P-gp in the brain may be a common cause underlying drug resistance in epilepsy, as increased P-gp expression has been found in brain tissues of medically intractable epilepsy patients with different pathological mechanisms, such as nervous system tumor, cortical dysplasia or hippocampal sclerosis [31–33]. Our previous studies have demonstrated that miRNAs could increase the concentration of antiepileptic drugs in drug-resistant HBMECs or in rat models of medically intractable epilepsy through regulating P-gp expression [16, 34].

Therefore, in the present study, we screened miRNAs which have been demonstrated to regulate P-gp expression in vitro for potential biomarkers of medically intractable epilepsy, in addition to the serum microarray assay. We also compared the diagnostic value of candidate miRNAs selected by the two approaches respectively. In the discovery phase, three miRNAs including miR-6514-3p, miR-6076-5p, and miR-6855-3p were identified as potential biomarkers for medically intractable epilepsy according to the results of microarray assay. In addition, miR-138-5p and miR-146a-5p were identified to regulate P-gp expression in drug-resistant HBMECs. In the validation phase, ROC curve analysis showed that the AUC values of miR-6514-3p and miR-6855-3p were lower than 0.7, suggesting limited value as peripheral biomarkers for medically intractable epilepsy. The AUC value of miR-301a-3p was 0.823, which was slightly lower than that reported previously [10]. This discrepancy may be caused by differences in group criteria, sample size and experiment methods. The AUC value of miR-138-5p and miR-146a-5p were higher than 0.85. Additionally, the AUC value of the miR-panel combining miR-138-5p and miR-146a-5p was higher than 0.9, which may have higher potentials as peripheral biomarkers of medically intractable epilepsy. Compared with the miRNAs selected by microarray assay, the miRNAs targeting P-gp had higher diagnostic value for medically intractable epilepsy. As the circulating miRNAs in the serum comes from a variety of sources, the results of microarray assay may be influenced by various factors and comorbid diseases. In fact, the gene annotation analysis indicated that the differentially expressed miRNAs in the serum miRNAs were involved in various biological processes and pathways, including nervous system development, signal transduction, material transport and so on. In this study, miR-138-5p and miR-146a-5p were revealed to regulate P-gp expression by modulating the activation of the NF-κB signaling pathway in vitro. Previous evidence has demonstrated that NF-κB is one of the key regulators of P-gp, due to the presence of NF-κB-binding sites in the promoter of P-gp [35]. Additionally, NF-κB is a converging site of various pathological processes like inflammation, oxidative stress and neurotransmitter dysregulation, which could further induce P-gp overexpression in the brain of epilepsy patients [36]. Previous studies have implied that the expression of P-gp and the activation of NF-κB were increased in the brain of epilepsy patients with hippocampal sclerosis. Our previous study has validated that miR-138-5p could directly bind the 3′-untranslated region (3′-UTR) of NF-κB p65 by dual-luciferase reporter assay [19]. In addition, previous study has demonstrated that miR-146a-5p was the key regulator that regulates the activity of the NF-κB signaling pathway in the negative feedback system. In detail, the expression of miR-146a-5p is upregulated by the activation of NF-κB through the TLR/MyD88-dependent pathway. While, overexpression of miR-146a-5p in turn inhibits activity of the NF-κB signaling pathway by directly inhibiting the expression of of IRAK1 and TRAF6, the upstream molecules of NF-κB [37, 38].

Although circulating miRNAs tend to be promising biomarkers for medically intractable epilepsy, there are still some limitations. The circulating miRNAs in the serum come from a variety of tissues, and it is hard to separate them using current technologies. Additionally, miRNAs, as important endogenous regulators, can target multiple genes and participate in various functions of the human body. Thus, the expression spectrum of miRNAs in the serum can be influenced by other diseases. Therefore, a comprehensive evaluation of other related diseases is needed to eliminate interference when using circulating miRNAs as diagnostic biomarkers for medically intractable epilepsy. Therefore, combing circulating miRNAs with auxiliary clinically relevant measurements, such as EEG and brain imaging, can improve the accuracy of diagnosis.

## Conclusions

**Fig. 6:**
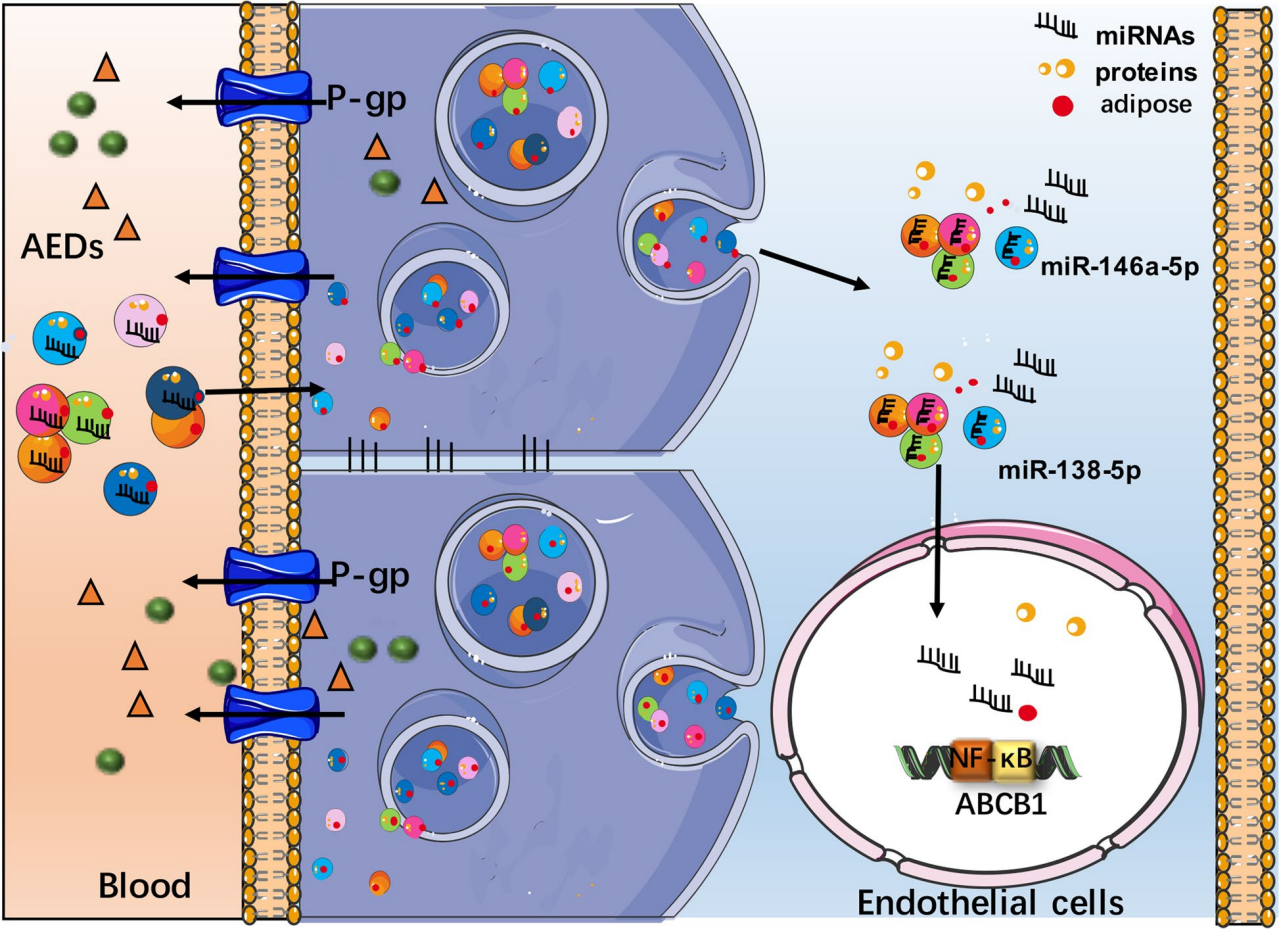
Illustration of the potential mechanism of circulating miRNAs targeting P-gp as peripheral biomarkers for medically intractable epilepsy

In this study, we found that the miR-138-5p and miR-146a-5p have high diagnostic value as peripheral biomarkers for medically intractable epilepsy, which might regulate P-gp expression via the NF-κB signaling pathway (Fig. 6). However, the number of cases with medically intractable epilepsy enrolled in this study was small, so future studies in a larger number of cases are needed to verify these results.

## Supplementary Information

**Additional file 1.**

**Additional file 2.**

**Additional file 3.**

**Additional file 4.**

**Additional file 5.**

## Data Availability

The datasets generated and analyzed in this study are available from the corresponding author on reasonable request.

## Abbreviations

ASMs: Antiseziure medications
AUC: Area under the curve
EEG: Electroencephalogram
GO: Gene ontology
HBMECs: Human brain microvascular endothelial cells
KEGG: Kyoto Encyclopedia of Genes and Genomes
NF-κB: Nuclear factor-kappa B
P-gp: P-glycoprotein
PHT: Phenytoin
ROC: Receiver operating characteristic
3′-UTR: 3′-untranslated region
