# Supplementary material for: Evaluation of P-glycoprotein-targeting circulating microRNAs as peripheral biomarkers for medically intractable epilepsy
Source: Acta Epileptol. 2023 Jan 23;5:5. doi: 10.1186/s42494-022-00116-x (PMC11960242; doi:10.1186/s42494-022-00116-x)
Supplement: Supplementary file 2 — Additional file 2. [file 42494_2022_116_MOESM2_ESM.pdf]

Profiling of microRNA in serum/plasma and other biofluids

Guidelines for the miRCURY LNA™ Universal RT microRNA PCR System, 3rd edition.

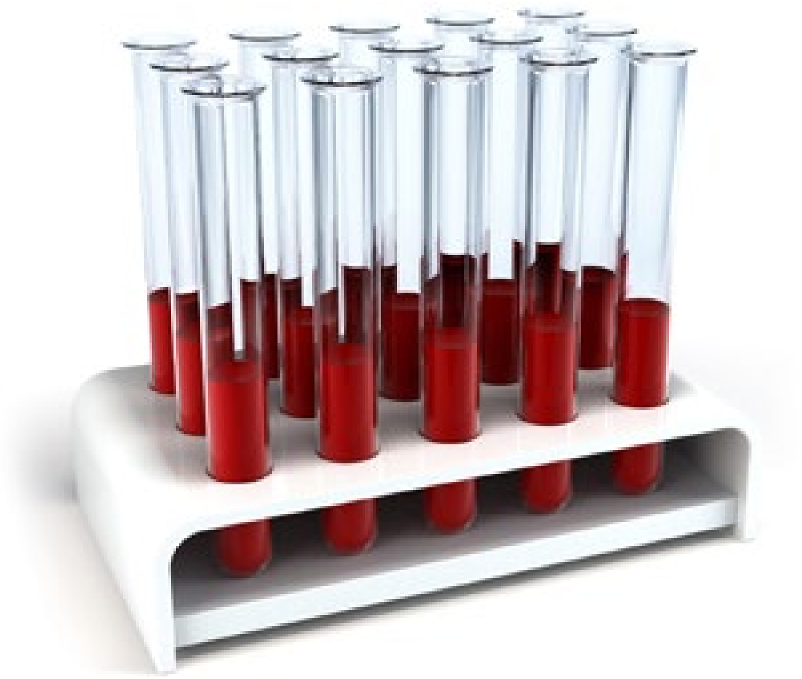

EXIQON
Seek Find Verify

www.exiqon.com

Table of Contents

Introduction 3

Challenges of microRNA profiling from serum/plasma and other biofluids. 3

How to choose between serum and plasma 3

Proper collection and preparation of samples 4

Optimal isolation of RNA 4

Conducting quality control of RNA 6

First strand cDNA synthesis 7

Real-Time PCR amplification 8

Normalization 9

Additional reading and resources 10

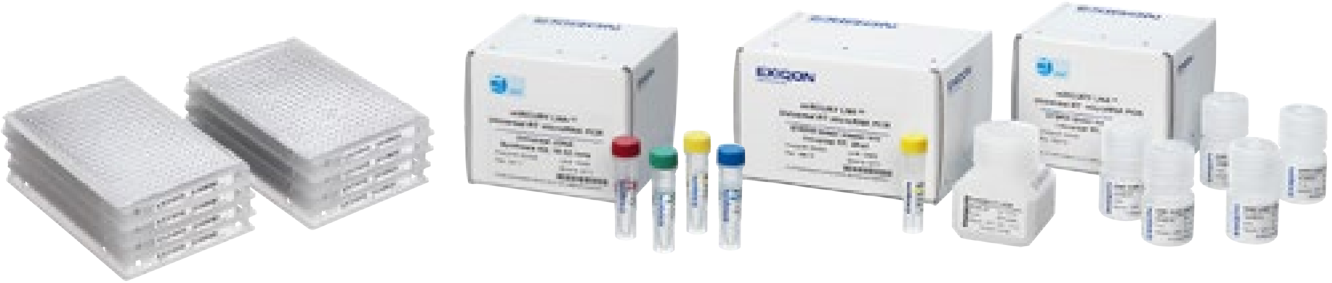

Introduction

MicroRNAs in serum/plasma and other biofluids hold great promise as minimally invasive diagnostic biomarkers for a wide range of diseases and biological processes. These short regulating RNAs have wide-ranging biological potential, are limited in number and are relatively stable in clinical samples such as serum/plasma, urine and other biofluids. However, microRNA profiling in biofluid samples is challenging and the development of robust biomarkers requires not only a highly sensitive and accurate microRNA detection method, but also optimized and standardized procedures for sample handling and preparation as well as reliable methods for sample and data QC.

Exiqon offers a range of products for highly sensitive, specific and robust detection of microRNAs in biofluids. Our strictly validated LNA™ enhanced qPCR assays offer the most sensitive and specific microRNA profiling available. Biofluid samples are challenging to work with in many ways. They contain low levels of RNA, high levels of inhibitors and are susceptible to many pre-analytical variables. To address these challenges, we have focused on developing highly reliable assays combined with extensive QC procedures. Our recommendations are based on many years' experience with both microRNA profiling and biomarker discovery and validation in biofluid samples. These guidelines focus on setting up microRNA profiling experiments from blood serum and plasma but contain useful information for microRNA experiments using other biofluids as well. The guidelines provide important information and tips to ensure successful microRNA quantification using the miRCURY LNA™ Universal RT microRNA PCR system.

Challenges of microRNA profiling from serum/plasma and other biofluids

MicroRNA profiling in biofluid samples holds great promise, but there are several challenges to overcome in order to successfully perform such experiments. These guidelines focus on serum and plasma, but many of the conclusions are valid for other biofluid samples.

First, plasma and serum and other biofluids contain very low amounts of RNA. This means that normal RNA quality control using Bioanalyzer or OD measurements is not suitable for these type of samples. Second, microRNAs detected in biofluids may have a cellular or an extracellular origin, the latter being the most interesting in terms of biomarker discovery. Avoiding cellular contamination and hemolysis (lysis of red blood cells) in serum/plasma samples, are therefore important challenges to overcome.

Third, biofluids contain inhibitors of the reverse transcriptase and polymerase enzymes used in the qPCR reaction. Minimizing the carry-over of qPCR inhibitors into the RNA sample as well as monitoring sample quality are important obstacles for consideration.

Finally, normalization of qPCR data from biofluid samples can be challenging because some of the larger small RNA species frequently used as reference genes (such as U6 RNA) are present in extremely low concentrations in serum and plasma as well as other biofluids. This means that great care must be taken when choosing controls for normalization. When working in biofluids like urine or cerebrospinal fluid (CSF), another challenge is the large variations in microRNA content between samples. Urine and CSF from healthy individuals typically contain much less microRNA compared to samples from diseased individuals or those exposed to drugs or toxins, which may contain significantly elevated levels of organ-specific microRNAs. In this document, we will present recommendations on ways to overcome these challenges.

How to choose between serum and plasma

RNA from both plasma and serum can be accurately profiled using the miRCURY LNA™ Universal RT microRNA PCR system. Both sample types have been used successfully for biomarker discovery. In general we observe slightly lower Cq values from plasma samples, which could be due to varying levels of thrombocyte contamination in the samples. On the other hand, differences in coagulation times and temperature may lead to variation between serum samples. Comparisons of normal serum and plasma indicate that there is less variation within a properly sampled serum dataset compared to a corresponding plasma dataset. Figure 1 shows a comparison between serum and plasma samples.

Figure 1. microRNA profiles from serum and plasma are very similar. Comparison of Cq values from 103 of the most commonly expressed microRNAs in serum and plasma.

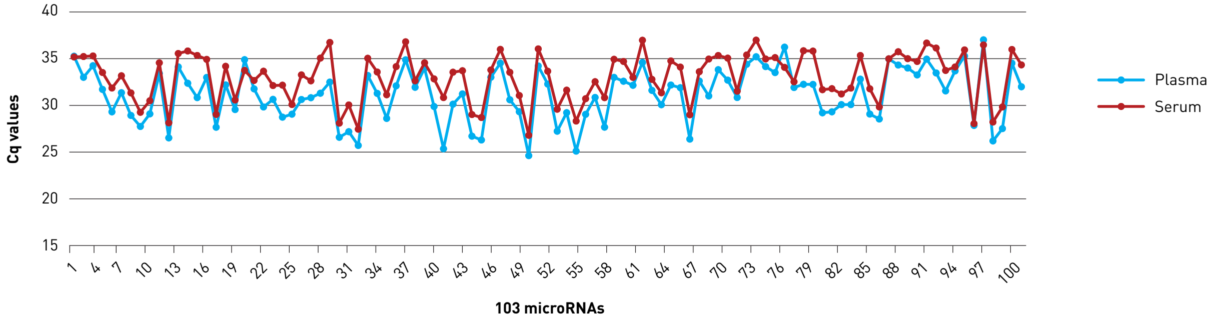

Proper collection and preparation of samples

Collection of whole blood is the first step in the preparation of plasma and serum. A method that preserves the RNA expression profile during and after collection of whole blood is important for accurate analysis of microRNA expression in blood plasma and serum. Use a well-described, standardized procedure for sampling to avoid introducing technical variation at this step. In addition, samples should be collected under similar conditions and where possible at the same time. When collecting new samples, it is recommended to process the whole blood immediately into either serum or plasma. We recommend the NCI's Early Detection Research Network (EDRN) standard operating procedures for the collection and preparation of serum and plasma. For optimal results from archived samples, care should be taken to select only samples that have been collected and processed according to the same protocol.

> Consistent and standardized sample collection and handling is essential

Figure 2. Suitability of different blood preparations for microRNA PCR analysis. Real-time PCR for miR-103-3p and miR-21-5p was performed using triplicate RT reactions on total RNA purified from either EDTA-plasma, citrate-plasma, heparin-plasma, or serum. Average Cq values for each triplicate are shown, demonstrating robust amplification from all sources except heparin-plasma.

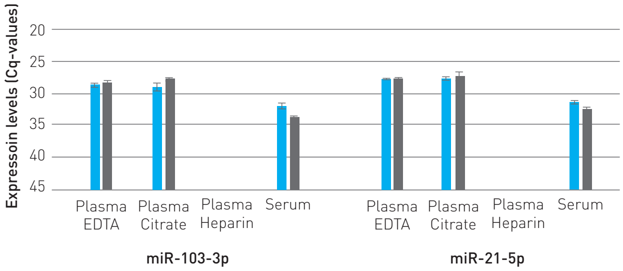

In all processing steps from whole blood to serum and plasma, measures should be taken to prevent lysis of cells, this is also important for other biofluid samples. Failure to do so may lead to contamination of the samples with RNA from intact cells. The RNA from intact cells could mask or prevent the subsequent detection of subtle changes in the microRNA expression profile. In the preparation of plasma, anticoagulants such as EDTA and sometimes citrate are normally used. These anticoagulants are both fine to use in contrast to heparin, which is known to inhibit downstream enzymatic steps such as cDNA synthesis and PCR (Figure 2). Currently, there is no reliable way of removing heparin from RNA samples or from the original blood serum/plasma samples. Therefore, AVOID HEPARIN in any of the processing steps of whole blood.

Once prepared, serum, plasma and other biofluid samples may be stored in RNase- free tubes (e.g. cryo-tubes) at -80° C. Alternatively, RNA isolation may be carried out immediately.

Optimal isolation of RNA

Working with RNA requires special precautions to prevent RNase contamination of the reagents and degradation of the RNA sample. The tips box below provides simple guidelines for good laboratory practice to ensure optimal performance of PCR experiments:

Tips: General guidelines for handling and storage of RNA

The following precautions should be taken to prevent RNase contamination and degradation of the RNA sample and reagents:

- Always wear disposable gloves, and work in a nuclease-free environment
- Use nuclease-free, low nucleic acid binding plastic ware and filter barrier pipette tips
- Keep tubes capped when possible, always spin tubes before opening
- For long-time storage, RNA may be stored at -80° C.
- Avoid repeated freeze-thaw cycles

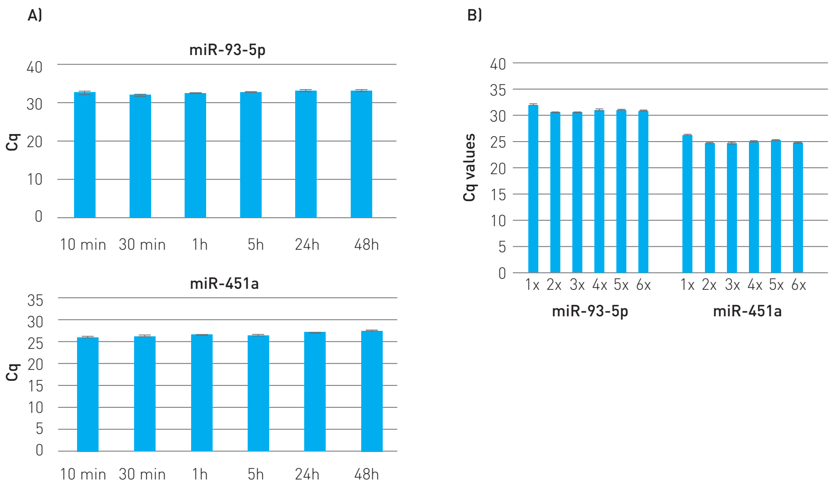

Figure 3. High stability of microRNA in EDTA-plasma samples. **A)** Real-time PCR was performed using triplicate RT reactions on total RNA purified from EDTA-plasma. The plasma was stored at room temperature before RNA extraction for the indicated times. No evidence of microRNA degradation in plasma was observed, even after prolonged storage of up to 48 hours at room temperature. **B)** Real-time PCR was performed using triplicate RT reactions on total RNA purified from EDTA-plasma. The plasma was submitted to 1-6 freeze-thaw cycles as indicated with no effect on microRNA amplification, demonstrating that plasma microRNA is stable towards multiple freeze-thaw cycles.

As mentioned earlier, biofluid samples contain only small amounts of RNA which means that there is a high risk that a significant proportion of the RNA is lost during extraction. For this reason, we recommend adding carrier RNA during the purification procedure, and subsequently using RNA amounts based on starting volume rather than RNA quantity based on optical measurements in downstream enzymatic steps. The use of a carrier ensures the highest and most consistent yield from serum and plasma samples (Figure 4). When selecting a carrier, it is important to choose a source which is guaranteed to be free from microRNAs. Such a source is RNA from the bacteriophage MS2 (available from Roche Applied Science cat. no. 10165948001), which is routinely used by Exiqon Services.

Figure 4. Carrier RNA improves RNA isolations from serum/plasma. Adding carrier to the RNA extraction increases reproducibility between isolations and leads to more consistent yields.

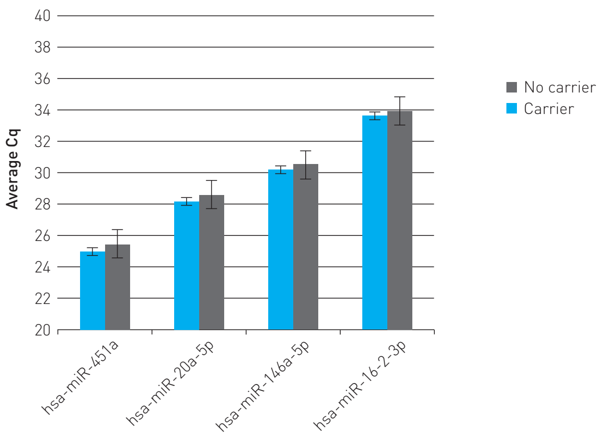

In addition to low amounts of RNA, biofluid samples also tend to contain high levels of enzyme inhibitors that can affect the efficiency of the reverse transcription or PCR reactions. Therefore it is important to choose a purification method which minimizes the carry-over of such inhibitors while maximizing the RNA yield.

Biofluid samples require optimal RNA isolation procedures

Exiqon offers an optimized kit for isolation of small RNA (<1000 bp) from serum/plasma and other biofluids: miRCURY™ RNA Isolation Kit - Biofluids (prod no. 300112 and 300113). Recommended input volumes depend on sample type and origin (see details in Table 1).

Table 1. Recommended starting volumes for different biofluids.

	Serum/plasma	Urine	CSF	Other biofluids*
Human samples	200 µL	200 µL	200 µL	200 µL
Rodent samples	50 µL**	200 µL	Not tested	50 µL**
RNA eluate for PCR***	4 µL	4 µL	8 µL	4 µL

*)For subsequent qPCR analysis, start volume may need to be adjusted to keep PCR inhibitors at a minimum.
**) Add RNase-free water to keep final volume at 200 µL.
***) Volume used as input in a 20 µL RT reaction using Exiqon's Universal cDNA Synthesis Kit.

Conducting quality control of RNA

Standard methods for measurement of the RNA yield and quality are inappropriate for use with biofluid samples. The presence of carrier RNA in these samples makes measuring the low levels of endogenous RNA by OD260 impossible. Even if carrier was not included during the isolation, the RNA concentration in the eluate would still be too low for reliable OD260 quantification on a NanoDrop or other spectrophotometers. Therefore, an alternative way of monitoring yield and normalizing sample input has to be used. Exiqon recommends using RNA amounts based on starting volume rather than RNA quantity (see below under cDNA synthesis).

To asses the quality of RNA isolated from the cell-free serum and plasma these parameters should be considered:

1. The efficiency of the extraction and the yield
2. The absence of any inhibitors of the cDNA synthesis and the PCR enzymes
3. The presence or absence of RNA resulting from cellular contamination or hemolysis
4. The presence of typical serum and plasma microRNAs

These parameters can be investigated using a combination of synthetic spike-in RNAs and selected endogenous microRNA assays. If working with other biofluid samples, microRNAs relevant for that type of sample need to be determined, either by literature study or a pilot study using a miRNome panel.

Exiqon has developed a set of synthetic RNA spike-ins that can be used to perform qPCR based quality control of RNA samples. The RNA spike-ins can be used to monitor the efficiency of RNA isolation, cDNA synthesis and PCR amplification (see Table 2 for details). The use of RNA spike-ins may also reveal potential presence of nucleases.

All spike-ins are available in the RNA spike-in kit and assays for the spike-ins are all present on Exiqon's ready-to-use microRNA PCR panels. In addition, a separate microRNA QC PCR panel is also available which contains a combination of 12 qPCR assays for the RNA-spike-ins and endogenous microRNAs. It is recommended that at least a subset of samples in larger studies are quality controlled in order to identify any potential problems in the sample set, before committing to microRNA profiling. Please refer to the miRCURY LNA™ Universal RT microRNA PCR, RNA spike-in kit and microRNA QC PCR panel manuals for details.

Table 2. Overview of assays in the microRNA QC PCR Panel and how they can be used to monitor the efficiency and yield of the RNA isolation, the performance of the cDNA synthesis and PCR reactions as well as general sample quality. For detailed information, please see the manuals for the microRNA QC PCR panel and the RNA Spike-in kit.

Assay name	Applications for serum/plasma samples
UniSp2, UniSp4 and UniSp5	• Three different concentrations<br>• Add to lysis buffer during RNA purification<br>• Use to monitor RNA isolation efficiency
UniSp6 and cel-miR-39-3p	• Different concentrations<br>• Add to cDNA synthesis reaction<br>• Check for RT and PCR inhibitors
UniSp3	• Template and primers present in PCR panels<br>• Independent PCR monitoring<br>• Use as Inter-plate calibrator, IPC
miR-451a and miR-23a-3p	• Unique Hemolysis indicator<br>• Check ΔCq(miR-23a-3p – miR-451a)
miR-30c-5p, miR-103-3p, miR-124-3p and miR-191-5p	• Biologically relevant, endogenous microRNAs<br>• Should be present in RNA from serum/plasma and some other biofluids<br>• Use for general sample quality check

UniSp2, UniSp4, UniSp5, and cel-miR-39-3p are part of the RNA Spike-in Kit.
The UniSp6 spike-in is provided with the Universal cDNA Synthesis Kit II and its corresponding primer set available in Exiqon's ExiLENT SYBR® Green master mix.

Tips: Successful RNA extraction and template preparation

The first critical step for successful expression profiling of microRNAs involves the purification and preparation of total RNA that includes small RNAs (<200 nt) from a biological sample. Therefore, the method used for RNA sample preparation is critical to the success of the experiment. The following points should be considered before starting the experiment:

- Always include a carrier RNA like MS2 RNA, in order to ensure the highest and most consistent yield from the samples
- The comparison of samples prepared using different RNA isolation methods is not recommended
- The isolation of RNA and the reaction steps preceding real-time PCR should be performed in rooms separate from where real-time PCR reactions are carried out in order to avoid contaminating the cDNA with PCR amplicons
- If working with plasma, make sure that EDTA or citrate was used as anticoagulant. Heparin is not suitable, when doing qPCR profiling
- Before isolating RNA from biofluids other than serum/plasma, consider whether to include the cellular fraction. For some sample types, like urine, performing separate analysis of the cell pellet and cell-free fraction may be relevant

QC check 1: Qualifying RNA for profiling

The amount of RNA that can be extracted as well as the amount of inhibitors left after extraction can vary from sample to sample. Before committing RNA samples to profiling on microRNA qPCR panels we recommend ascertaining the purification yield and absence of PCR inhibitors by testing different RNA sample input amounts in the cDNA synthesis reaction (e.g. 0.5 µL, 1.0 µL, 2.0 µL, and 4.0 µL in a 10 µL RT reaction) with assays for the RNA-spike-ins and a few microRNA assays. This is easily done using the microRNA QC PCR panel. Samples with low RNA yield can be excluded from further studies based on low signal in this pre-study, and samples containing PCR inhibitors will show dilution curves without the expected linear relationship between sample input and signal (Figure 5). Good microRNAs for this pre-study include the following microRNAs which are typically detected at medium to high levels:

Serum/plasma:
- hsa-miR-103-3p
- hsa-miR-191-5p
- hsa-miR-423-3p
- hsa-miR-451a

Urine:
- hsa-miR-30c-5p

CSF:
- hsa-miR-124-3p

Often, one or more of these microRNA assays may also be good for the normalization of expression data, but the stability of their expression needs to be determined (see below). The results of the dilution curve analysis should solely be used to assess the quality of the obtained RNA. We do not recommend adjusting the sample input volume in the RT for the qPCR panel analysis based on these results. As mentioned above, we recommend including the same volume of purified RNA in the RT for all serum/plasma samples.

Figure 5. Optimizing RNA input amounts. Performing an RNA dilution series can be used to determine that input amounts are within the linear range of the system, and to ensure a good level of signal, with no sign of inhibition (e.g. Sample A at 8 µL). Increasing the RNA volume to increase signal levels can have the opposite effect and result in less signal due to carry-over of more inhibitory compounds into the RT-PCR reaction (e.g. Sample B at 8 µL).

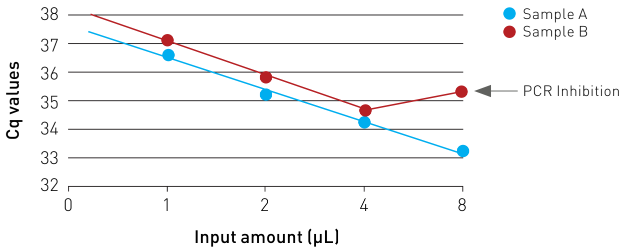

QC check 2: Post-profiling analysis of cell contamination

The circulating, or extracellular microRNAs, are the most interesting targets for microRNA profiling in serum and plasma. An overrepresentation of RNA species from white or red blood cells in the profile may be an indication that cells have lysed at some point prior to the RNA isolation. This may be due to incomplete removal of white blood cells and platelets from the sample, and/or due to hemolysis. The presence of cellular RNA species may disturb the serum/plasma microRNA profiling experiment resulting in a distorted and non-reproducible profile. Monitoring hemolysis in serum and plasma samples can be done by various spectrophotometric methods that measure free hemoglobin levels. However, if the RNA has already been extracted or the original sample is no longer available, an alternative strategy would be to compare the level of a microRNA highly expressed in red blood cells (hsa-miR-451a), with a microRNA unaffected by hemolysis (hsa-miR-23a-3p)[2]. We found that delta Cq (miR-23a-3p - miR-451a) is a good measure of the degree of hemolysis where values of more than five is an indicator of possible erythrocyte microRNA contamination, and a delta Cq of 7–8 or more indicates a high risk of hemolysis affecting the data obtained in human samples (the values are different in mouse and rat samples). It is important to note that many microRNAs in serum and plasma are not affected by hemolysis and therefore it may still be possible to detect disease associated microRNA biomarkers even from samples affected by hemolysis. However it is crucial to be aware of the possible effects on the microRNA profile when performing normalization and data analysis so that any systematic bias can be eliminated.

First strand cDNA synthesis

The quality control of RNA described above will indicate whether inhibitors in general are present in the RNA samples. To minimize or alleviate the effect of smaller variations in inhibitor content between samples, it is recommended to perform cDNA synthesis in a larger reaction volume than for other samples (see Figure 6 and the Instruction Manual for miRCURY LNA™ Universal RT microRNA PCR for serum/plasma samples at www.exiqon.com/mirna-pcr).

Tips: How to avoid hemolysis

- Use good and consistent sample collection device throughout study (e.g. BD Vacutainer)
- Follow manufacturer's instructions!
- Avoid drawing blood from a hematoma
- Avoid frothing of the sample
- Make sure the venipuncture site is dry
- Avoid a probing, traumatic venipuncture
- Avoid prolonged tourniquet application or fist clenching
- Use correct size needle (~22 gauge)
- Vacuum tubes should be filled completely

2. Blondal et al. (2012), Methods. 59(1):164-9

Figure 6. Guide for experimental set up. The figure displays a scalable experimental set up overview that fits all project sizes, from initial screening to validation.

Sample Type	Serum, plasma or other biofluids				
Panels or primer sets	miRNome panel I	miRNome panel I+II	Pick-&-Mix/Focus panel: 1-96 assays	Pick-&-Mix/Focus panel: 97-192 assays	Individual assays (<96)
Universal cDNA reactions per kit	16	8	64	32	64
Universal cDNA reaction volume (μL)	40	80	10	20	10
Dilution of cDNA in ExiLENT Master Mix	50x				40x
ExiLENT Master Mix consumption (μL)	2000	4000	5/assay		

Real-Time PCR amplification

Dilution of the cDNA synthesis reaction is a standard procedure conducted prior to PCR when using the miRCURY LNA™ Universal RT microRNA PCR system. Due to the larger RT reaction volume used for biofluid samples, the dilution factor of the cDNA is reduced relative to normal samples (see Figure 6).

Figure 7 shows the high reproducibility between different reverse transcription reactions on RNA from serum that can be achieved using the above reaction volumes. From this point on, all handling and PCR cycling conditions follow procedures described in the Instruction Manual for miRCURY LNA™ Universal RT microRNA PCR for biofluid samples.

For a general overview of which products Exiqon offer for microRNA profiling in biofluids and when to introduce the different spike-ins, please see Figure 8.

Figure 7. Excellent reproducibility between RT reactions on total RNA from serum. Raw Cq values from two separate RT reactions (RT1 and RT2) on total RNA purified from 65 μL serum are shown. A total of 730 microRNAs were profiled. Only microRNAs with Cq values below 35 have been included (133 data points).

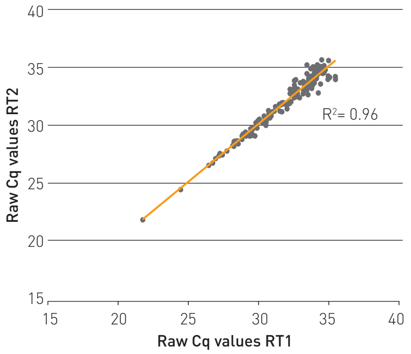

ROX:
The ExiLENT SYBR® Green master mix does not include ROX passive reference dye. This may be needed for some instruments.

ABI instruments:
Use manual baseline and threshold settings. SDS template files for this and with pre-defined plate layout can be downloaded at www.exiqon.com/sds

Normalization

The purpose of normalization is to remove technical variation in data which is not related to the biological changes under investigation. Proper normalization is critical for the correct analysis and interpretation of results from real-time PCR experiments. The most commonly used methods for normalization are:

1. To identify and use stably expressed reference genes.
2. To use the mean expression value of all commonly expressed microRNAs in a given sample as normalization factor[3].

Exiqon's GenEx software supports both of these approaches and it is recommended to investigate which of these methods will provide the best normalization of the data in question.

Biofluid samples typically do not contain the larger small RNA species, e.g., 5S, U6 and snoRNAs, that are sometimes used for normalization in other samples. Another difference is that often the number of microRNAs detected in biofluid samples is rather low. When using mean expression values for normalization, a high number of microRNAs need to be expressed. Therefore, as a general guideline, consider to identify and use stably expressed reference genes for normalization if the number of detected microRNAs is below 80-100.

When using stably expressed reference genes for normalization, it is recommended to test several control candidates (reference genes) before setting up the actual microRNA expression analysis. These candidates should be chosen among genes that can be expected to be stably expressed over the entire range of samples being investigated.

When working with serum and plasma the candidates are typically chosen based on the literature or pre-existing data (e.g. qPCR panel screening). Hsa-miR-93-5p, hsa-miR-103-5p, hsa-miR-191-5p, hsa-miR-423-3p, and hsa-miR-425a are available on Exiqon's Serum/plasma Focus microRNA PCR Panel as candidate reference genes. These are typically medium to highly expressed and may be stably expressed, but their potential use as reference genes needs to be evaluated on a study-to-study basis.

For other biofluid samples we recommend to empirically determine the best reference microRNAs by doing an initial pilot study and use geNorm or NormFinder from the GenEx software to identify the best candidates. For further details regarding experimental set up and data analysis of microRNA qPCR experiments we refer to our microRNA qPCR guidelines available here:http://www.exiqon.com/ls/Documents/Scientific/miRNA-qPCR-guidelines.pdf

GenEx Software
For fast and easy data analysis. Learn more at www.exiqon.com/qpcr-software

3. Mestdagh et al. (2009), Genome Biol, 10, R64

Figure 8. Biofluids sample processing guide

Sample Type	Serum or plasma	Urine	CSF	Other biofluids
Sample preparation	miRCURY™ RNA Isolation Kit – Biofluids and RNA Spike-in kit, UniRT (UniSp2, 4 and 5 to monitor sample isolation efficiency)			
cDNA synthesis	Universal cDNA Synthesis Kit and RNA Spike-in kit, UniRT (UniSp6 and cel-miR-39-3p to monitor cDNA and qPCR efficiency)			
RNA QC (recommended)	miRCURY™ microRNA QC PCR Panel	miRCURY™ microRNA QC PCR Panel	Spike-in control primer sets	miRCURY™ microRNA QC PCR Panel or Spike-in control primer sets
Initial screening	Serum/Plasma Focus microRNA PCR panel	Toxicology Focus or miRNome microRNA PCR panels on a few samples	miRNome microRNA PCR panels on a few samples	miRNome microRNA PCR panels on a few samples
Profiling	Serum/Plasma Focus microRNA PCR panel	Toxicology Focus or Pick-&-Mix microRNA PCR panel with selected relevant assays	Pick-&-Mix microRNA PCR panel with selected relevant assays	Pick-&-Mix microRNA PCR panel with selected relevant assays

Additional reading and resources

Find more information in our biofluids reading room: **www.exiqon.com/biofluids**

The following publications can be downloaded from **www.exiqon.com/mirna-pcr**

- Instruction Manual for microRNA LNA™ Universal RT microRNA PCR serum and plasma
- Instruction Manual for miRCURY™ RNA Isolation Kit - Biofluids
- Instruction manual for miRCURY™ microRNA QC PCR Panel
- Pitfalls and recommendations for microRNA expression analysis using qPCR - guidelines to setting up microRNA qPCR
- SDS template files for ABI instruments
- Exiqon GenEx software

Data analysis software and tools are described in more details at **www.exiqon.com/qPCR-resources**

EDRN Standard Operating Procedures can be found here: **http://edrn.nci.nih.gov/resources/standard-operating-procedures**

www.exiqon.com/e-talk displays presentations about the miRCURY LNA™ Universal RT microRNA PCR system and the advantages of using the LNA™ technology

Exiqon, LNA™, and miRCURY™ are registered trademarks of Exiqon A/S, Vedbaek, Denmark. Locked Nucleic Acids (LNA™) are covered by patents and patent applications owned by Exiqon A/S. SYBR® Green is a registered trademark of Invitrogen. Concerning miRCURY LNA™ Universal RT microRNA PCR: NOTICE TO PURCHASER: LIMITED LICENSE Purchase of this product includes an immunity from suit under patents specified in the product insert to use only the amount purchased for the purchaser's own internal research. No other patent rights are conveyed expressly, by implication, or by estoppel. Further information on purchasing licenses may be obtained by contacting the Director of Licensing, Applied Biosystems, 850 Lincoln Centre Drive, Foster City, California 94404, USA.

For life science research use only. Not for use in diagnostic procedures.

Contact information
Outside North America
Phone: +45 45 65 09 29 · Fax: +45 45 66 18 88
North America
Phone: +1 781 376 4150 · Fax: +1 781 376 4152

www.exiqon.com

EXIQON
Seek Find Verify

919102 - 03/2013
